# Supplementary material for: Structural basis of ion – substrate coupling in the Na+-dependent dicarboxylate transporter VcINDY
Source: Nat Commun. 2022 May 12;13:2644. doi: 10.1038/s41467-022-30406-4 (PMC9098524; doi:10.1038/s41467-022-30406-4)
Supplement: Supplementary file 1 — Supplementary Information [file 41467_2022_30406_MOESM1_ESM.pdf]

Supplementary information for

Structural basis of ion – substrate coupling

in the Na^+-dependent dicarboxylate transporter VcINDY

David B. Sauer[1,2,&], Jennifer J. Marden[1,2], Joseph C. Sudar[2], Jinmei Song[1,2],

Christopher Mulligan[3]* and Da-Neng Wang[1,2]*

[1] Department of Cell Biology, New York University School of Medicine, New York, NY 10016, USA

[2] Skirball Institute of Biomolecular Medicine, New York University School of Medicine, New York, NY 10016, USA

[3] School of Biosciences, University of Kent, Canterbury, Kent, UK

*Correspondence to: C.M. (c.mulligan@kent.ac.uk), D.N.W. (da-neng.wang@med.nyu.edu)

[&]: Current address: Centre for Medicines Discovery, Nuffield Department of Medicine, University of Oxford, Oxford, UK

Supplementary Table 1. Cryo-EM data collection and structure determination of NaCT

	VcINDY-Na^+ (300 mM)	VcINDY-*apo*
**EMPIAR ID**	10969	10970
**EMDB ID**	25757	25756
**PDB ID**	7T9G	7T9F
Magnification (x)	105,000	105,000
Voltage (kV)	300	300
Number of movies	4,253	4,514
Electron dose (e^-/Å^2)	65	65
Defocus range (µm)	-0.5 – -2.0	-0.5 – -2.5
Collection mode	Super-resolution	Super-resolution
Effective pixel size (Å)	0.415	0.415
**<u>Data processing</u>**		
Initial number of particles	1,520,631	6,100,049
Final number of particles	144,865	134,038
Symmetry imposed	C2	C2
Map resolution* (Å)	2.83	3.23
**<u>Model refinement</u>**		
Non-hydrogen atoms	6,691	6,682
Protein residues	890	890
Na^+, H_2O	4, 5	-
Mean B factor		
Protein (Å^2)	44.09	80.40
Na^+, H_2O (Å^2)	40.92, 34.86	-
RMS deviations		
Bond lengths (Å)	0.003	0.004
Bond angles (°)	0.550	0.841
Molprobity score	1.58	1.52
Clash score	5.49	5.27
Poor rotamers (%)	0.00	0.28
Ramachandran plot		
Favored (%)	95.94	96.39
Allowed (%)	4.06	3.61
Outliers (%)	0.00	0.00
Model resolution† (Å)	2.8	3.2

* Map resolution determined by Gold-Standard FSC threshold of 0.143.
† Resolution cutoff at which the model and sharpened map Fourier coefficients reach 0.143.

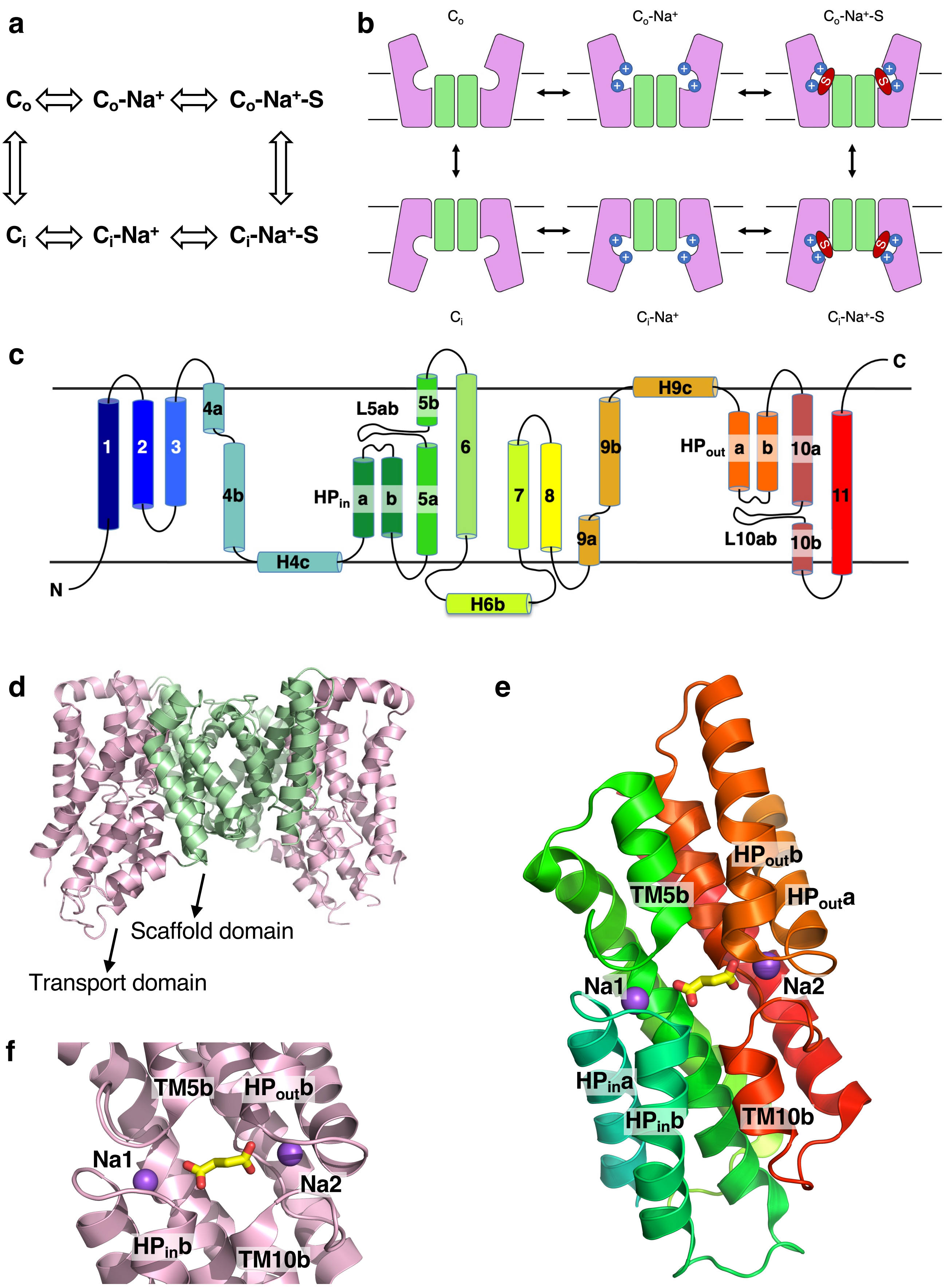
a
C_o ⇔ C_o-Na^+ ⇔ C_o-Na^+-S
C_i ⇔ C_i-Na^+ ⇔ C_i-Na^+-S
b
C_o
C_o-Na^+
C_o-Na^+-S
C_i
C_i-Na^+
C_i-Na^+-S
c
N
C
1
2
3
4a
4b
H4c
L5ab
5b
5a
HP$_{in}$ a b
6
H6b
7
8
9a
9b
H9c
HP$_{out}$ a b
10a
L10ab
10b
11
d
Scaffold domain
Transport domain
e
HP$_{out}$b
HP$_{out}$a
TM5b
Na1
Na2
HP$_{in}$a
HP$_{in}$b
TM10b
f
TM5b
HP$_{out}$b
Na1
Na2
HP$_{in}$b
TM10b

Supplementary Fig. 1. **Kinetic cycle of VcINDY and molecular structure of its C_i-Na^+-S state.** **a,** Kinetic cycle and, **b,** schematic model of VcINDY in its transport cycle. C_o: outward-facing conformation; C_i: inward-facing conformation; S: substrate. The number of co-transported Na^+ for VcINDY is 3, but only two are shown here. All available biochemistry evidence indicates that sodium ions bind before and release after the substrate. **c,** Transmembrane topology of VcINDY. **d,** X-ray structure of VcINDY dimer. Each protomer consists of a scaffold domain and a transport domain. **e-f,** Structure of the transport domain (e) substrate and sodium binding sites (f) (PDB ID: 5UL7).

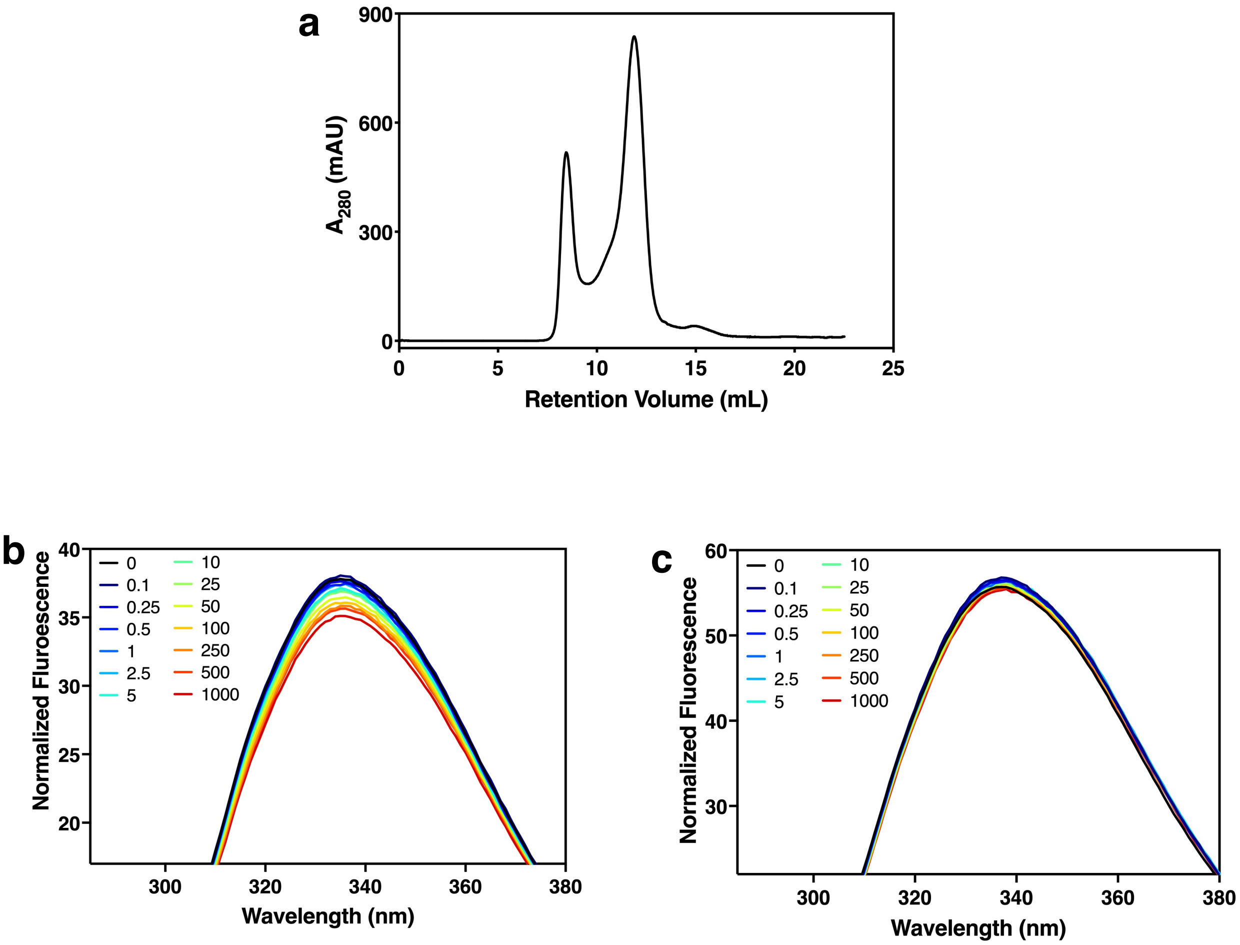

Supplementary Fig. 2. **Purification and substrate binding measurements of VcINDY.** **a,** Size-exclusion chromatography trace of VcINDY in dodecyl-maltoside detergent in the presence of 100 mM ChCl. Ch^+ instead of Na^+ was used as a cation to keep the protein stable. **b,** Measurements of succinate binding to detergent-purified VcINDY in the presence of 100 mM NaCl, using intrinsic tryptophan fluorescence quenching ($N = 4$). The K_d was determined to be 92.2 ± 47.4 μM. **c,** Measurements of succinate binding to VcINDY in the presence of 100 mM ChCl ($N = 4$). There was no binding that could be measured. In both **b** and **c**, the succinate concentrations in the legend are in μM.

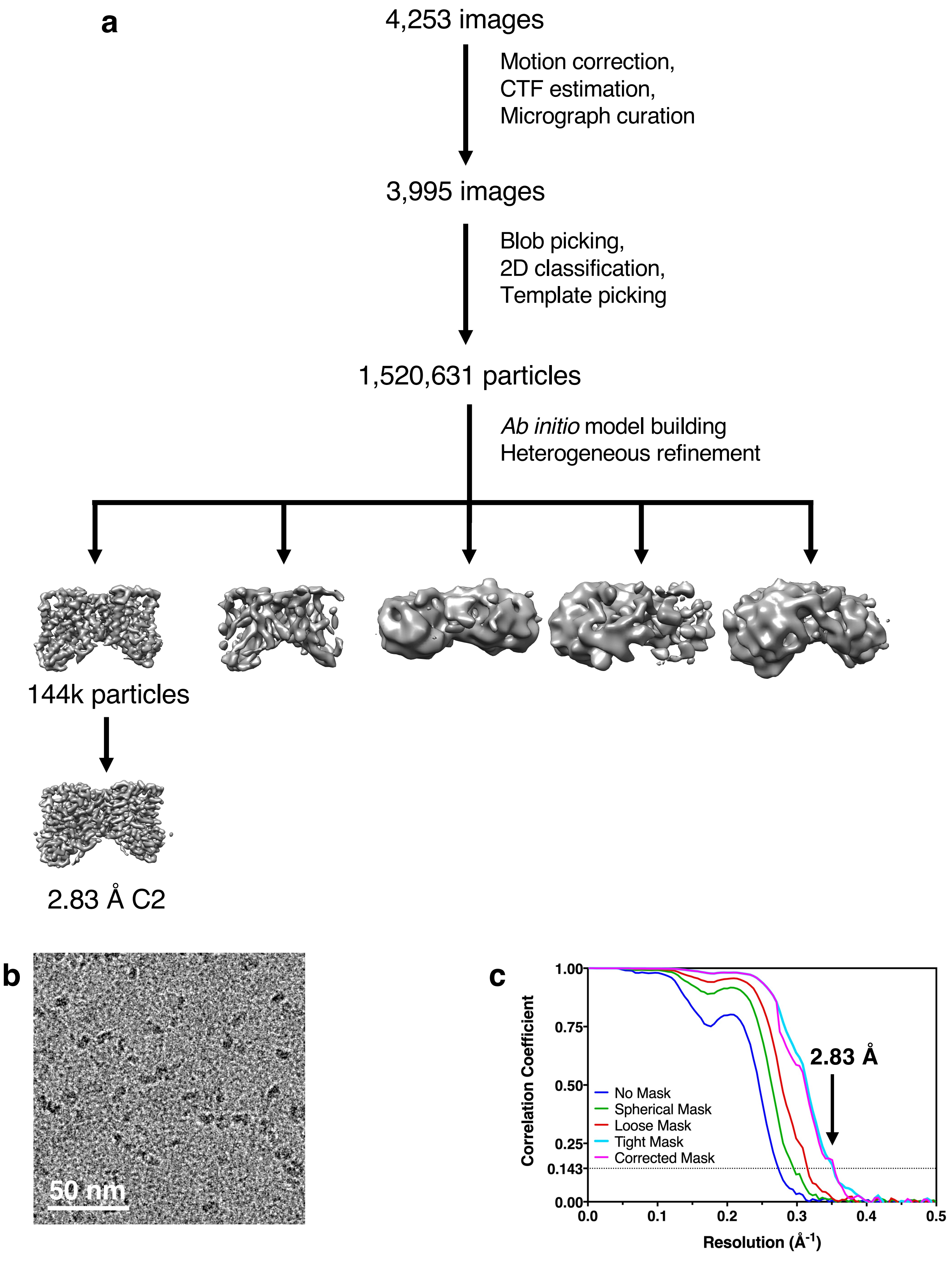

Supplementary Fig. 3. Cryo-EM structure determination of VcINDY in its C_i-Na^+ state solved in 300 mM Na^+. a, Workflow of cryo-EM structure determination of VcINDY structure in 300 mM Na^+. **b,** Cryo-EM micrograph. **c,** Fourier shell correlation curve. The gold-standard FSC resolution is indicated by the arrow.

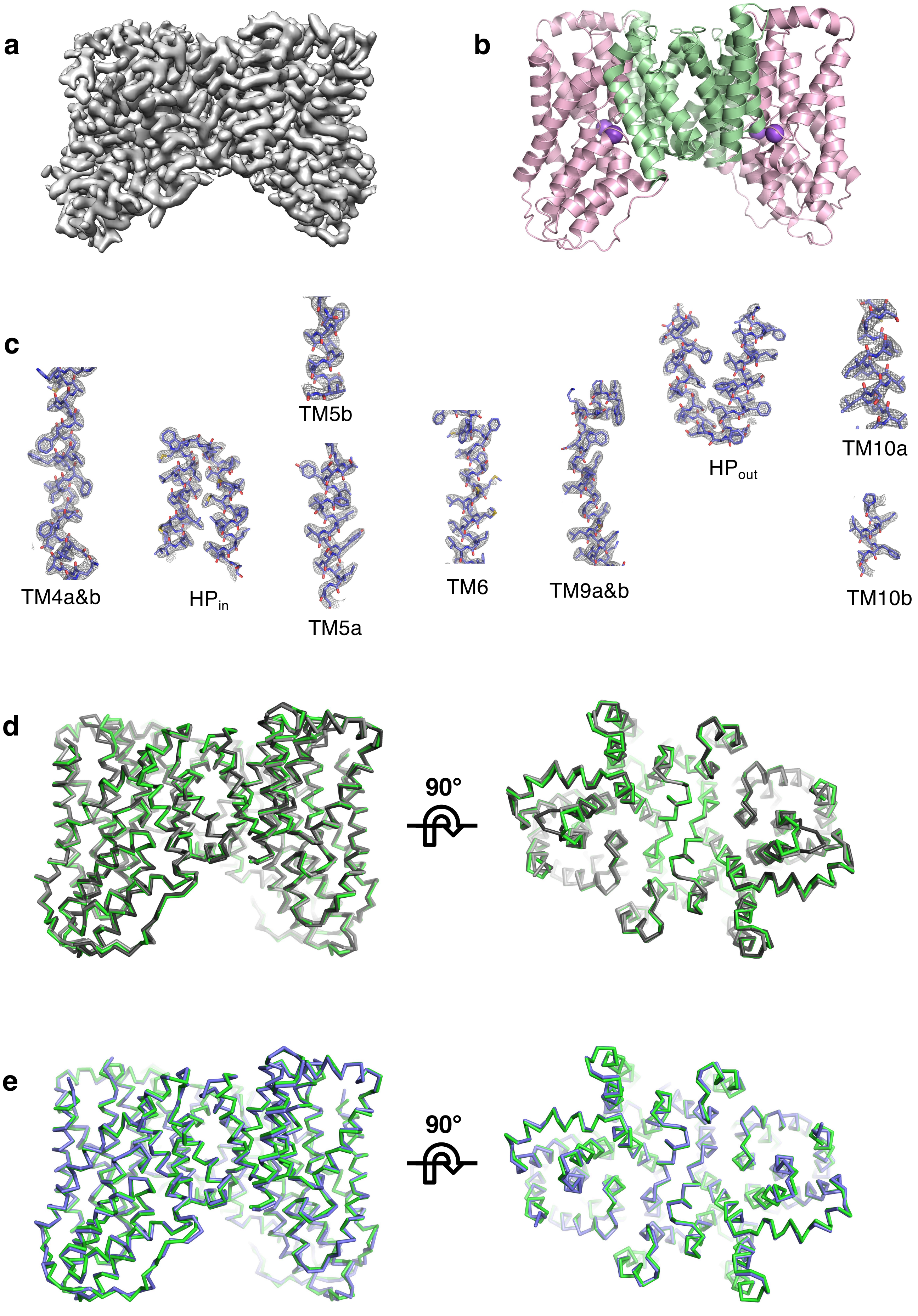

a
b
c
TM4a&b
HP$_{in}$
TM5b
TM5a
TM6
TM9a&b
HP$_{out}$
TM10a
TM10b
d
90°
e
90°

Supplementary Fig. 4. **Cryo-EM structure of VcINDY in its C_i-Na^+ state solved in 300 mM Na^+. a,** Cryo-EM map at 2.83 Å resolution obtained in 300 mM NaCl, contoured at 5.1 σ. **b,** Model of VcINDY in its C_i-Na^+ state. The scaffold domain and the transport domain in each protomer are colored green and pink, respectively. **c,** Cryo-EM densities of individual helixes showing the quality of the model to map fitting, contoured at 8.5 to 9.5 σ. **d,** Overlay of the VcINDY structure determined in 300 mM Na^+ (green) with two structures previously-determined in the presence of 100 mM Na^+, with Fab bound in nanodisc (PDB ID: 6WW5; light grey) and without Fab but in amphipol (PDB ID: 6WU3; dark grey). Left, viewed from within the membrane plane. Right, viewed from the periplasmic space. **e,** Overlay of the VcINDY structure in the C_i-Na^+ state determined in 300 mM Na^+ (green) with X-ray structure of VcINDY in the C_i-Na^+-S state (PDB ID: 5UL7; blue).

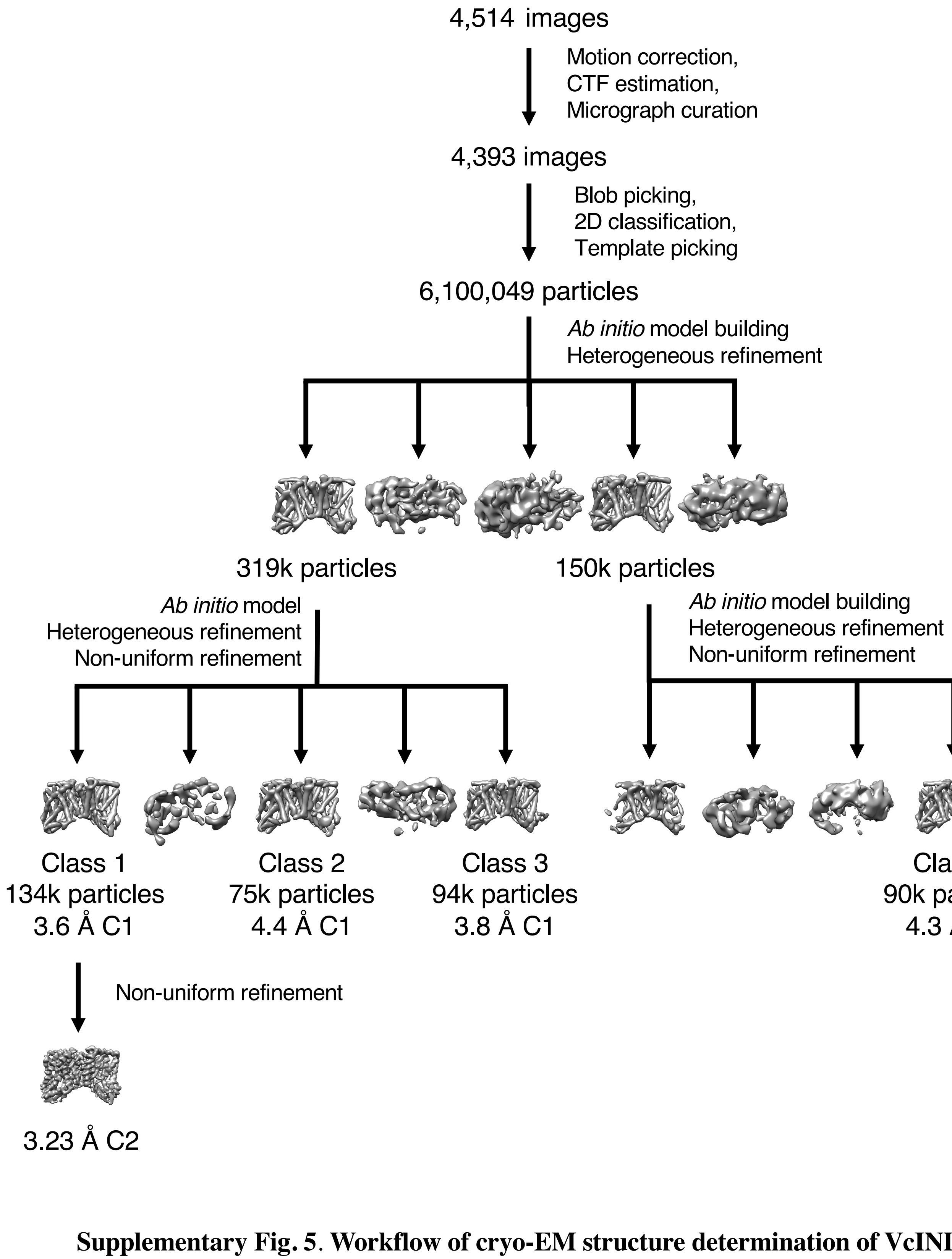

Supplementary Fig. 5. Workflow of cryo-EM structure determination of VcINDY in C_i-*apo* state in choline.

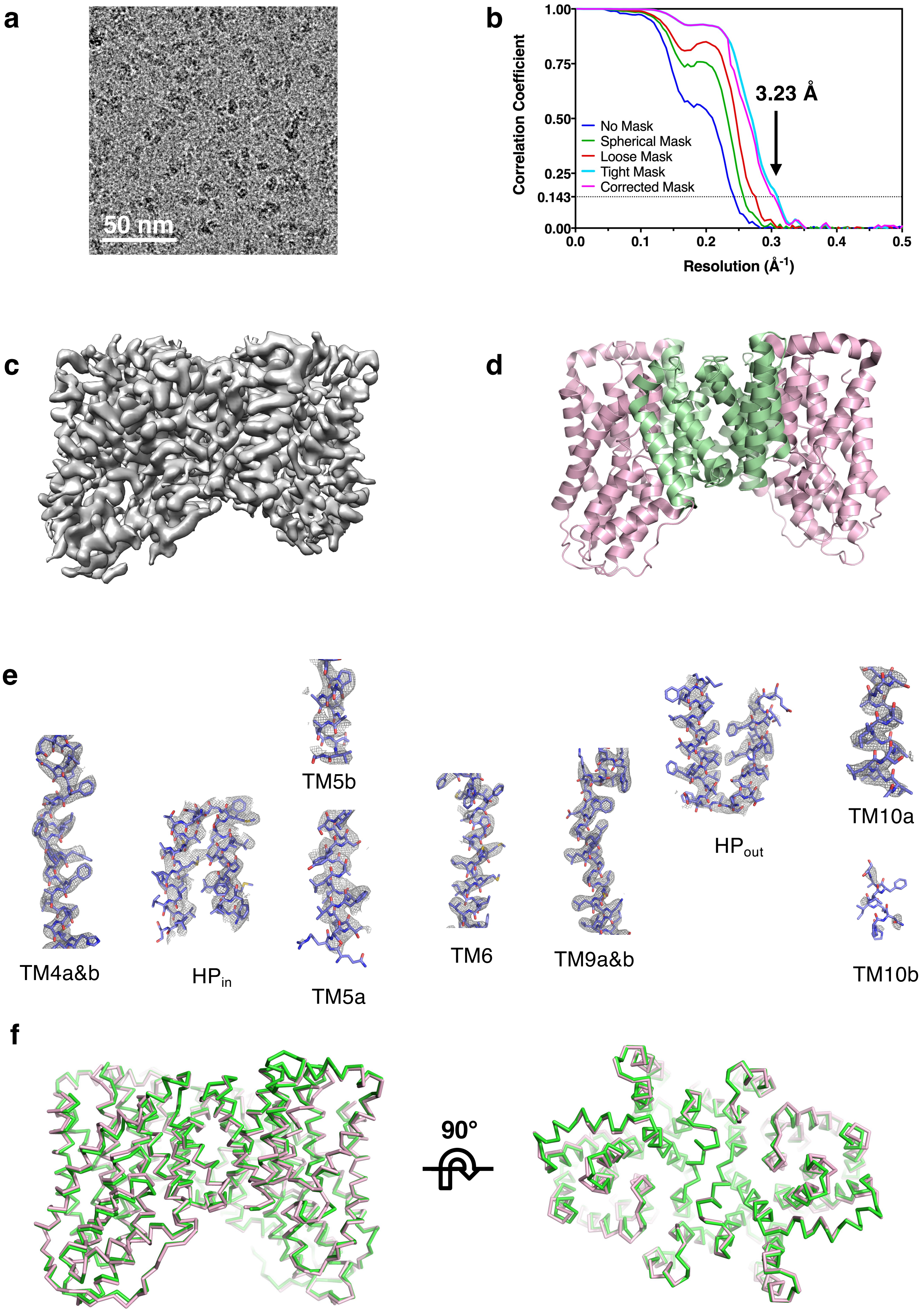

a
50 nm
b
1.00
0.75
0.50
0.25
0.143
0.00
Correlation Coefficient
0.0
0.1
0.2
0.3
0.4
0.5
Resolution (Å^{-1})
No Mask
Spherical Mask
Loose Mask
Tight Mask
Corrected Mask
3.23 Å
c
d
e
TM4a&b
HP$_{in}$
TM5b
TM5a
TM6
TM9a&b
HP$_{out}$
TM10a
TM10b
f
90°

Supplementary Fig. 6. Cryo-EM structure determination of VcINDY in its C_i-*apo* state solved in Ch^+. **a,** Cryo-EM micrograph of VcINDY prepared in 100 mM Choline chloride. **b,** Fourier shell correlation curve. The gold-standard FSC resolution is indicated by the arrow. **c,** Cryo-EM map at 3.23 Å resolution obtained in Ch^+, contoured at 4.8 σ. **d,** Model of VcINDY in its C_i-*apo* state. The scaffold domain and the transport domain in each protomer are colored green and pink, respectively. **e,** Cryo-EM densities of individual helixes showing the quality of the model to map fitting, contoured at 5.5 to 8.5 σ. **f,** Overlay of the VcINDY structure in its C_i-Na^+ state determined in Na^+ (green) with that of the C_i-*apo* state determined in Ch^+ (light pink). Left, viewed from within the membrane plane. Right, viewed from the periplasmic space.

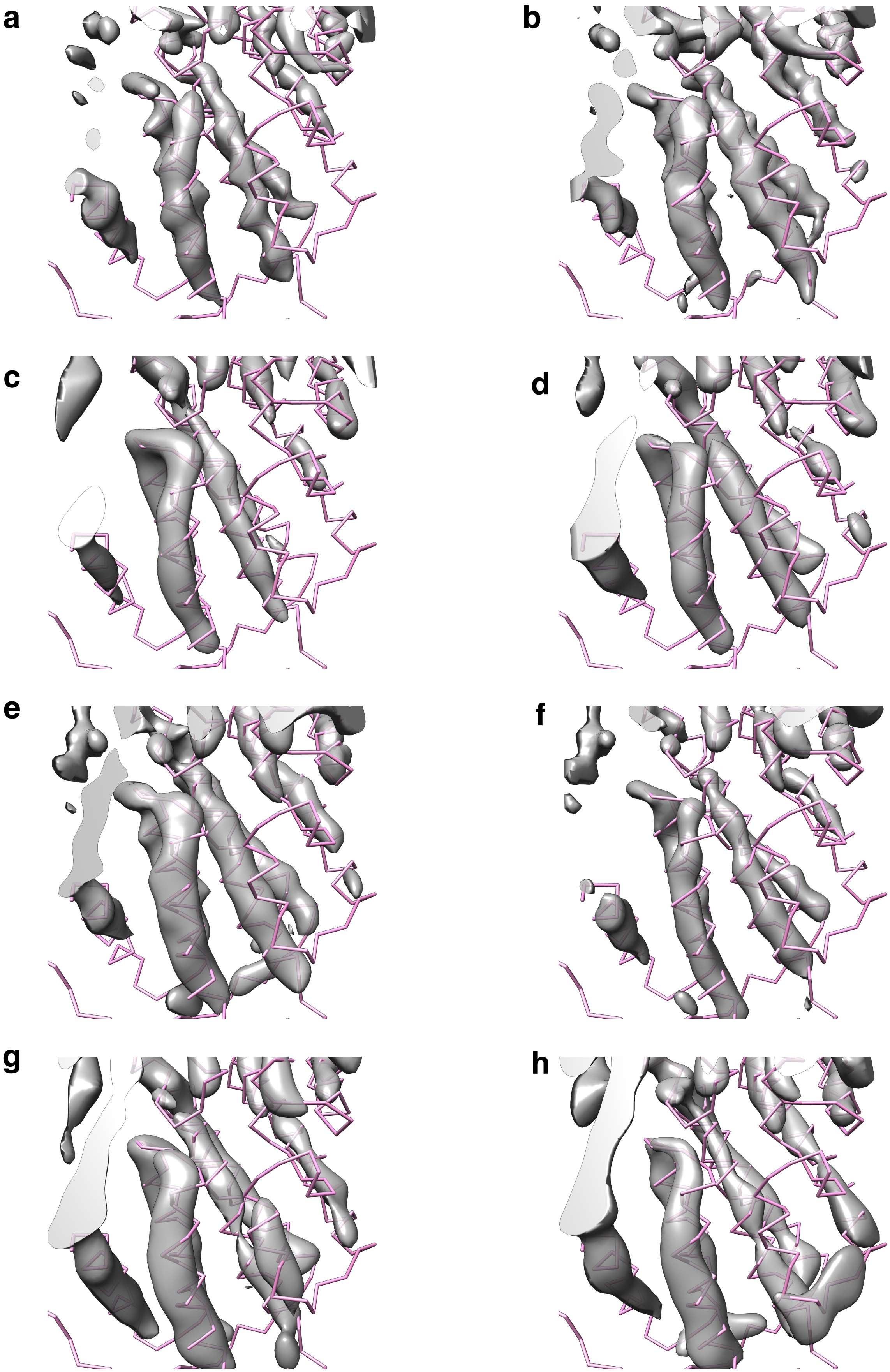
a
b
c
d
e
f
g
h

Supplementary Fig. 7. **Cryo-EM density of VcINDY in 100 mM ChCl.** Unsharpened Cryo-EM maps of the transport domains processed with C1 symmetry for each protomer of VcINDY prepared in 100 mM choline chloride. **a, b,** Class 1, **c, d,** Class 2, **e, f,** Class 3, and **g, h,** Class 4. The refined VcINDY-*apo* Cα model is shown in pink. Maps are contoured such that the corresponding scaffold domains have equal volume.

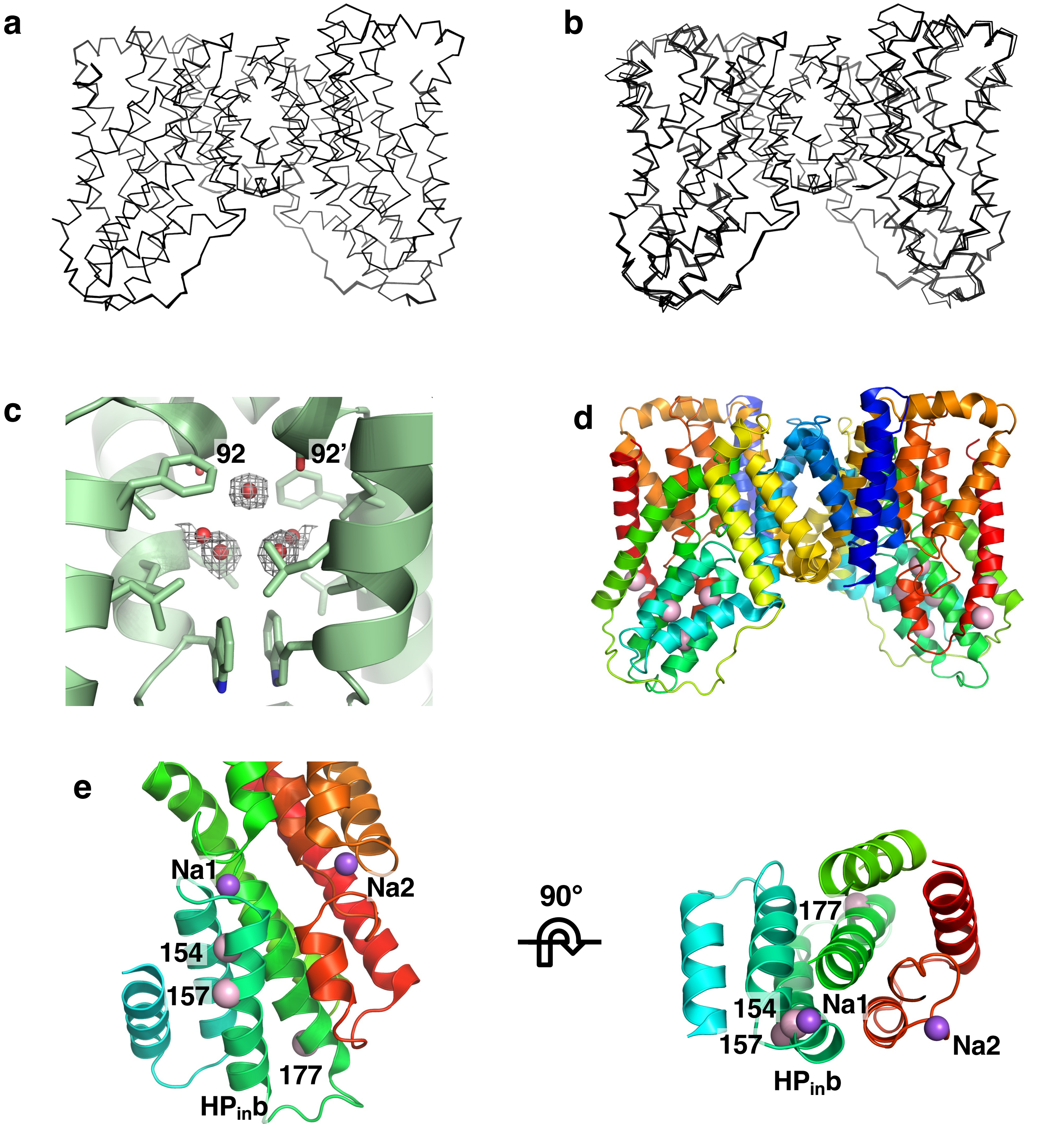

Supplementary Fig. 8. **Flexibility of VcINDY-*apo* structure. a,** NMR-style analysis of the VcINDY structure in Na^+. **b,** Simulated annealing analysis of the VcINDY structure in choline$^+$. The resolution limit for refinement in **a** and **b** was truncated to 3.23 Å. **c,** Water molecules observed in the VcINDY cryo-EM map in the C_i-Na^+ state. Coulomb potential map show contoured at 3.5 σ. **d,** VcINDY dimer structure with positions of the residues labeled in the current work indicated by pink balls on the C_i-Na^+ state structure. **e,** Positions of relevant residues labeled in the previous work (Sampson, et al., J. Biol Chem, 2020, 295, 18524 – 18538).
