# Supplementary figures and images for: Structural basis of ion – substrate coupling in the Na+-dependent dicarboxylate transporter VcINDY
Source: Nat Commun. 2022 May 12;13:2644. doi: 10.1038/s41467-022-30406-4 (PMC9098524; doi:10.1038/s41467-022-30406-4)

Cysless

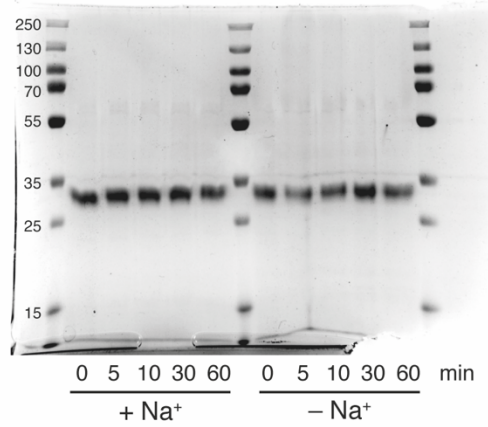

A155C

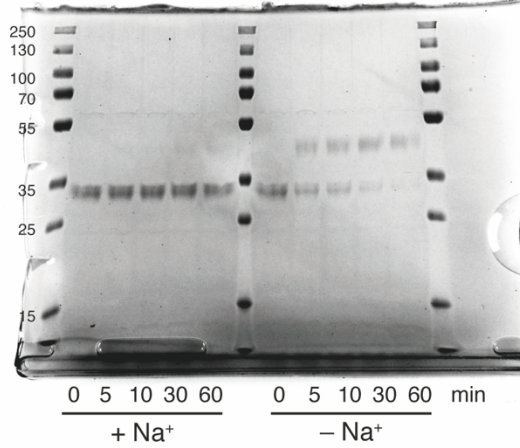

A189C

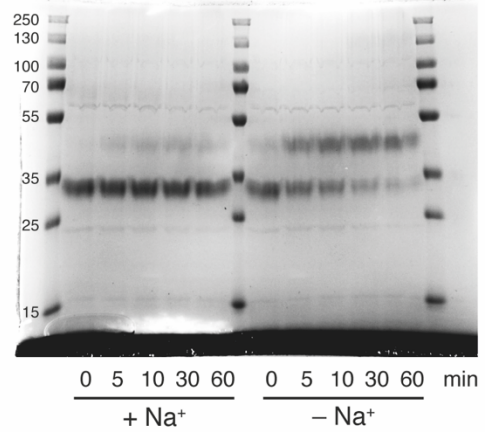

V162C

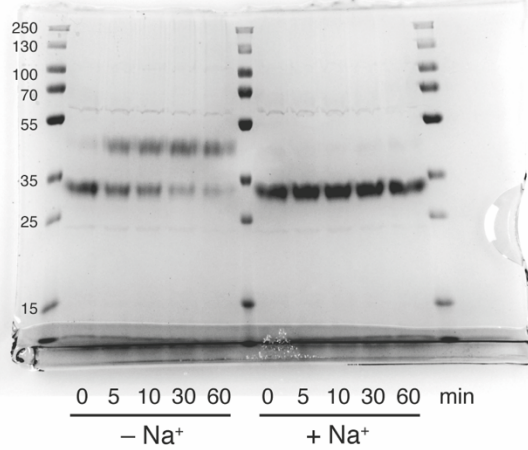

L138C

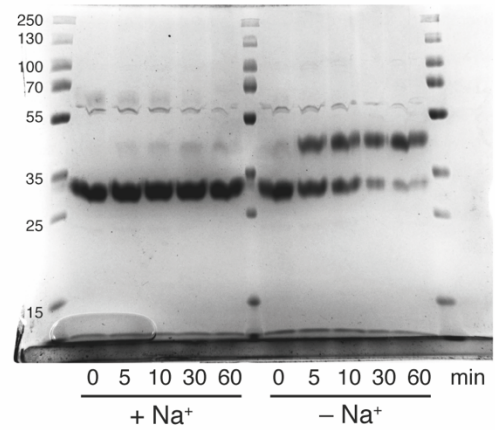

S436C

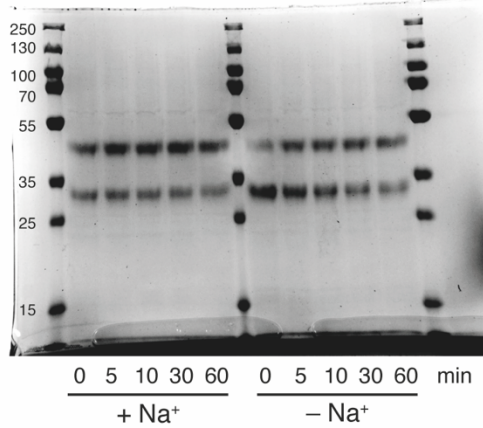

V441C

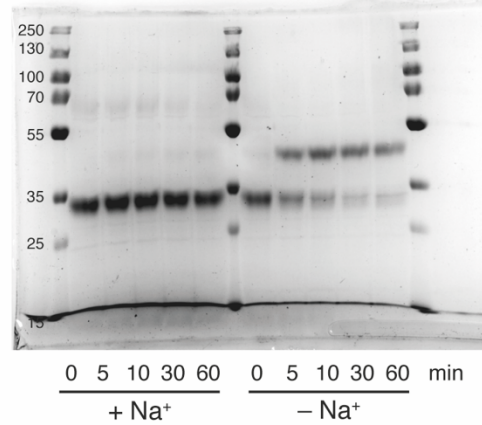

Supplement: Supplementary file 4 — Source Data [file 41467_2022_30406_MOESM4_ESM.pdf]
